# Evaluation of the Antibacterial and Antifungal Potential of *Peltophorum africanum*: Toxicological Effect on Human Chang Liver Cell Line

**DOI:** 10.1155/2013/878735

**Published:** 2013-03-11

**Authors:** Benjamin I. Okeleye, Noxolo T. Mkwetshana, Roland N. Ndip

**Affiliations:** ^1^Microbial Pathogenicity and Molecular Epidemiology Research Group, Department of Biochemistry & Microbiology, Faculty of Science and Agriculture, University of Fort Hare, Private Bag X1314, Alice 570, South Africa; ^2^Department of Microbiology and Parasitology, Faculty of Science, University of Buea, P.O. Box 63, Buea, Cameroon

## Abstract

We assessed the *in vitro* antimicrobial activity of *Peltophorum africanum* by means of the agar well and macrodilution methods. The toxicity on a normal human liver cell (Chang liver cell) was determined using the CellTiter-Blue cell viability assay, and the compounds contained in the fractions were identified using GC-MS. Zone diameter of inhibition of the extract ranged from 12.5 ± 0.7  to  32 ± 2.8 mm for bacteria and from  7.5 ± 0.7  to  26.4 ± 3.4 mm for yeast. Marked activity of the extract was observed against *Plesiomonas shigelloides* ATCC 51903, with MIC and MLC values of 0.15625 and 0.3125 mg/mL, respectively. The extract was both bactericidal (MIC_index_ ≤ 2) and bacteriostatic/fungistatic (MIC_index_ > 2) in activity. Lethal dose at 50 (LD_50_) showed 82.64 ± 1.40 degree of toxicity at 24 hrs, and 95 percentile of cell death dose activity ranged from log 3.12 ± 0.01  to  4.59 ± 0.03. The activity of the eight fractions tested ranged from 1.0 ± 0.5  to  3.7 ± 1.6 mg/mL (IC_50_) and from  2.1 ± 0.8  to  6.25 ± 0 mg/mL (IC_90_). The extract was toxic to human Chang liver cell lines.

## 1. Introduction

Following the invention of modern medicine, herbal medicine suffered a setback, but recent advances in photochemistry and identification of plant compounds that are effective against life-threatening diseases have enhanced interest in it. In many developing countries, traditional medicine is one of the primary healthcare systems [1, 2]. Extracts and bioactive compounds with known antimicrobial activities isolated from plants can be of great impact in the formulation of new drugs, and the potential of higher plants as source for new drugs is still largely unexplored [3]. The increasing prevalence of multidrug-resistant microbial strains due to indiscriminate use of broad-spectrum antibiotics, immunosuppressive agent, and continuous epidemics of HIV infection adds urgency to the search for alternative treatment. Most horrible human diseases are caused by bacteria, viruses, fungi, and parasitic worms [4]. *Staphylococcus aureus*, a Gram-positive bacterium and an opportunistic pathogen of the human skin, has been incriminated in wound infection, toxic shock syndrome, and food poisoning [5], while *Pseudomonas aeruginosa* is a pathogen associated with pyogenic and urinary tract infections [6]. *Plesiomonas shigelloides* infections occur in the summer months and correlate with environmental contamination of freshwater and have been implicated in gastroenteritis [7]. Fungi especially *Candida *sp. and *Cryptococcus *sp. are increasingly being recognized as major pathogens in critically ill patients [8]. 

*Peltophorum africanum* (Sond Fabaceae), an African wattle (isiKhaba-mkhombe in Xhoza and Musese in Venda) is a semideciduous to deciduous trees, traditionally used in South Africa to alleviate gastric problems, human immunodeficiency virus/acquired immune deficiency syndrome (HIV/AIDS), and infertility [9]. Though the ethyl acetate extract of the stem bark of *P. africanum* has been previously investigated for its antimicrobial activity against clinical strains of *Helicobacter pylori* and other pathogens; [9] there is a dearth of information on its toxicity. This study was therefore aimed at elucidating the probable compounds responsible for the antimicrobial activity of the plant extract as well as evaluating its safety in an effort to validate its folkloric use in the treatment of microbial infections. 

## 2. Experimental Part 

### 2.1. Plant Extract

The ethyl acetate extract of the stem bark of *P. africanum* was selected based on the remarkable activity reported in our previous study [9]. The extract was prepared as described in our previous study with modifications. Briefly, dried powder of the stem bark (200 g) was extracted with 96% ethyl acetate (800 mL) and filtered after 48 hrs. The plant residue was reextracted exhaustively (three filtration processes), and the filtrate was concentrated on a rotary evaporator (Strike 202 Steroglass, Italy) at 70°C to remove the ethyl acetate. Fresh working stock of the extract was prepared by sterilizing in 100% DMSO for each bioassay analysis. The extract was aseptically bottled using Acrodisc 25 mm PF Syringe (Pall, USA) and then tested for sterility by putting 0.5 mL of the extract into 2.5 mL of nutrient broth. A sterile extract was indicated by a clear broth (absence of turbidity) after incubation at 37°C for 24 hrs. The extracts were kept at 4°C until use. 

### 2.2. Test Organisms

The microorganisms used were obtained from our microbial stock collection in the Department of Biochemistry and Microbiology University of Fort Hare, South Africa. The bacteria included *Staphylococcus aureus* NCTC 6571, *Pseudomonas aeruginosa* ATCC 15442, *Plesiomonas shigelloides* ATCC 51903, *Helicobacter pylori* ATCC 43526, *Streptococcus pyogenes* ATCC 49399, *Aeromonas hydrophila* ATCC 35654, *Shigella sonnei* ATCC 29930, and *Salmonella Typhimurium* ATCC 13311. The fungi included *Aspergillus flavus* ATCC 204304, *Aspergillus niger* ATCC 16888, *Candida albicans* ATCC 2091, and *Cryptococcus neoformans* ATCC 66031. All bacteria and fungi cultures were subcultured thrice for purity. The fungi were inoculated in Sabouraud dextrose broth and bacteria into nutrient broth (*H. pylori* was inoculated in brain heart infusion broth with Skirrow's supplement and 10% horse serum) and incubated for 24 hrs at 37°C (*H. pylori* was incubated microaerophilically in anaerobic jar with gas pack). The turbidity of the culture was adjusted with sterile saline solution to match 0.5 McFarland standards. 

### 2.3. Cell Line Growth and Maintenance

The human Chang liver cell line used in this study was a kind donation from Professor Maryna van de Venter of Nelson Mandela Metropolitan University, South Africa. Briefly, vials containing cells were taken from liquid nitrogen stocks. The cells were thawed in a water bath (37°C) and transferred to a 25 mm^3^ culture flask (TPP, Switzerland). A 1 mL thawed cell stock was diluted with 9 mL prewarmed Dulbecco's Minimum Essential Medium (DMEM) containing 10% fetal bovine serum (FBS). The cells were incubated in a 37°C humidified incubator (Shel Lab, USA), 5% CO_2_ for multiplication and adherence. Maintenance of cells was achieved by splitting the cells until the desired cell number and confluence was reached. 

### 2.4. Antimicrobial Assay

The agar well diffusion method was used as previously described [10, 11]. Agar plates were prepared using sterile brain heart infusion agar (Oxoid, England) with Skirrow's supplement and 10% horse serum for *H. pylori*, Mueller-Hinton (MH) agar (Merck, Gauteng, South Africa) for bacteria and potato dextrose agar (PDA) (Lab M, UK) for fungi. Strains of standardized cultures were evenly spread onto the surface of the agar plates using sterile swab sticks. Wells were punched in the plates using a sterile stainless 6 mm cork borer. The wells were filled with 100 *μ*L of 25 mg/mL, 50 mg/mL, and 100 mg/mL of the extract. Ten percent DMSO was used as a negative control and 30 *μ*g/ mL of amoxicillin and tetracycline as positive controls. Diffusion of the extracts, antibiotics, and DMSO was allowed at room temperature for 30 mins in a laminar flow cabinet and then incubated at 37°C (fungi, 27°C) for 24–72 hrs. Each experiment was conducted in duplicate, and the zone diameters of inhibition (mean ± SD) produced by each of the concentrations of the solutions were measured in millimeters and interpreted according to the Clinical and Laboratory Standards Institute (CLSI) guideline [12]. 

### 2.5. Minimum Inhibitory Concentration (MIC) Determination

The MIC was determined according to the method of Delahaye et al. [13] with modification. Different concentrations (0.005–10.0 mg/mL) of the extract were prepared by twofold serial dilutions in brain heart infusion broth (Oxoid, England) with Skirrow's supplement and 10% horse serum, Mueller-Hinton broth (Merck, Gauteng, South Africa) and sabouraud dextrose broth (Lab M, UK) for *H. pylori*, other bacterial strains, and fungi, respectively. Each tube was inoculated with 100 *μ*L of each of the adjusted microbial strain and incubated at 37°C (fungi, 27°C) for 24–72 hrs. Tetracycline and amoxicillin (30 *μ*g/mL) were used as positive controls, and two tubes each containing medium with/without a microbial strain and treatment were used as negative control. After the incubation period, the first tube in the series of concentrations that showed no visible trace of growth was taken as the MIC. 

### 2.6. Minimum Lethal Concentration (MLC) Assay

Fresh nutrient agar (Merck, Gauteng, South Africa), PDA (Lab M, UK), and Columbia blood agar (Oxoid, England) plates prepared for bacteria, fungi, and *H. pylori*, respectively, were inoculated with one loopful of culture taken from each of the MIC tubes for the determination of minimum bactericidal concentration (MBC) and minimum fungicidal concentration (MFC). After the incubation periods, the lowest concentration of the extract that did not produce any or >90% bacterial or fungal growth on the solid medium was regarded as the minimum lethal concentration (MLC) of the extract. The mechanism of antibiosis of the extracts was calculated using the ratio of MLC/MIC. When the ratio of MLC/MIC_index_ was ≤2, the extract was considered as bactericidal/fungicidal otherwise as bacteriostatic/fungistatic. If the ratio was ≥16 the extract was considered as ineffective [13, 14]. 

### 2.7. Assay for Cytotoxicity

Toxicity of the extract was evaluated on human Chang liver cell lines using microculture CellTiter-Blue viability (Promega, USA) assay [15]. For the assay, 96-well microplates were seeded with 100 *μ*L DMEM + high glucose, L-glutamine and sodium pyruvate (Thermo Sceintific, South Logan, Utah, USA) containing 3.0 × 10^3^ cells in suspension and incubated in a CO_2_ incubator regulated at 37°C and 5% CO_2_. After 24 hrs incubation and attachment, the cells were treated with 1000, 500, 250, 125, 75, 25, and 5 *μ*g/mL concentration of the extract. Exactly 60 *μ*M of curcumin (Sigma-Aldrich, South Africa) was used as positive control and 0.1% DMSO as negative control. After 24, 48, and 72 hrs of incubation, cell viability was determined by adding CellTiter-Blue as an indicator and further incubated for 4 hrs. Fluorescence was read at 570/620 nm using Analytical & Diagnostic Product Gen spectrophotometer (BioTek, USA). 

### 2.8. Fractionation of the Extract and Antimicrobial Activity of the Fractions

The ethyl acetate extract of *P. africanum *was fractionated using two solvent systems; toluene/ethanol (TEt; 90 : 5) and benzene/ethanol/ammonium hydroxide (BEtA; 90 : 10 : 1) using thin layer chromatography (TLC) and column chromatography methods [16, 17]. The inhibitory effect of the fractions was carried out in accordance with our previously reported method [11]. Briefly, twofold dilutions of the fractions (0.049–6.25 mg/mL) were prepared in 96-well plates containing brain heart infusion broth (Oxoid, England) with Skirrow's supplement and 10% horse serum, Mueller-Hinton broth (Merck, Gauteng, South Africa), sabouraud dextrose broth (Lab M, UK) for *H. pylori*, other bacteria, and yeast cell, respectively. Exactly 25 *μ*L of each strain (0.5 McFarland standards) was added into the wells and the assay performed in duplicate. After incubation at 37°C for 24 hrs (bacteria) and 27°C for 3 days (yeast), the absorbance was read at 620 nm with Analytical & Diagnostic Product Gen spectrophotometer (BioTek, USA). Concentration at which 50% (IC_50_) and 90% (IC_90_) of microbial growth were inhibited was determined. 

### 2.9. GC-MS Analysis for the Identification of Compounds in the Fractions

The fractions were dissolved and injected onto a GC column and helium was used as the carrier gas with injection volume of 1 *μ*L. Injection temperature and MS transfer were set at 280°C with acquisition mode scanning mass range of 40 to 550 m/z (electron ionization at 70 Ev). Agilent 6890N GC with CTC Combi-PAL Autosampler and Agilent 5975B MS with Rtx-5MS (30 m, 0.25 mm ID, 0.5 *μ*m film thickness) Restek 12723-127 were used for the analysis [18].

**Ethical Consideration**

This study which is a continuation of our line of studies on microbial pathogens and anti-infective from medicinal plants had been approved by the institutional review board of the University of Fort Hare.

## 3. Results 

### 3.1. Susceptibility and Resistant Phenotype

The ethyl acetate extract of *P. africanum* was tested against pathogenic bacteria, yeast, and mold in this study. All the eight bacteria and two yeast tested were susceptible at the three concentrations of 25, 50, and 100 mg/mL, with the zone diameter of inhibition ranging from 12.5 ± 0.7 to 32 ± 2.8 mm. For yeast, it ranged from 7.5 ± 0.7 to 26.4 ± 3.4 mm, while, for mold, no activity was observed. *Helicobacter pylori *ATCC 43526, *Streptococcus pyogenes* ATCC 49399, *Shigella sonnei* ATCC 29930, *Salmonella Typhimurium* ATCC 13311, *Aspergillus flavus* ATCC 204304*, Aspergillus niger *ATCC 16888, and *Cryptococcus neoformans *ATCC 66031 were resistant against tetracycline (positive control antibiotic) at 30 *μ*g/mL (Table 1). 

### 3.2. Mechanism of Antibiosis

The extract had bactericidal effect (MLC/MIC_index_ ≤ 2) on *P. aeruginosa*, *P. shigelloides*, *A. hydrophila*, *S. aureus*, and *S. Typhimurium*. Bacteriostatic/fungistatic effect (MLC/MIC_index_ > 2) was noted against *H. pylori*, *S. pyogenes*, *S. sonnei*, and *C. albicans*. Marked activity was observed against *P. shigelloides* with MIC and MLC values of 0.15625 and 0.3125 mg/mL, respectively (Table 2). 

### 3.3. Cytotoxicity Effect

We observed that at 5, 25, 50, 75, and 95 percentile of cell death after 24, 48, and 72 hrs of incubation, the extract dose activity ranged from log⁡⁡to  0.75 ± 0.03 − 1.05 ± 0.01, 0.82 ± 0.02 to 1.66 ± 0.01, 1.92 ± 0.01 to 2.15 ± 0.00, 2.51 ± 0.00 to 3.01 ± 0.01, and 3.12 ± 0.01 to 4.59 ± 0.03, respectively (Table 3). Lethal dose at 50 (LD_50_) showed 82.64 ± 1.40, 140.09 ± 1.33, and 121.07 ± 0.92 *μ*g/mL degree of toxicity at 24, 48 and 72 hrs, respectively (Table 4). 

### 3.4. *In Vitro* Antimicrobial Activity of the Fractions from the Ethyl Acetate Extract of *P. africanum*

Activity of the fractions on the bacteria and yeast cells are shown in Table 5. Fractions BEtA2, BEtA4, T1, and BEtA3 inhibited 50% of test organisms (IC_50_) at 1.0 ± 0.5, 1.0 ± 0.6, 1.6 ± 1.7, and 1.7 ± 0.9 (mg/mL), meanwhile 90% were inhibited (IC_90_) at 2.5 ± 0.8, 3.1 ± 2.3, 2.1 ± 0.8, and 3.8 ± 1.9 (mg/mL), respectively. Of the four fractions, the best activity against yeast (*C. albicans*) was demonstrated by BEtA4 with IC_50_ and IC_90_ values of 0.391 and 1.563 mg/mL, respectively, while for the bacteria, the same fraction exhibited marked activity against *P. aeruginosa* with IC_50_ and IC_90_ of 0.024 and 0.097 mg/mL, respectively. 

### 3.5. Identified Compounds in the Fractions of Ethyl Acetate Extract of *P. africanum*

**Figure 1:**
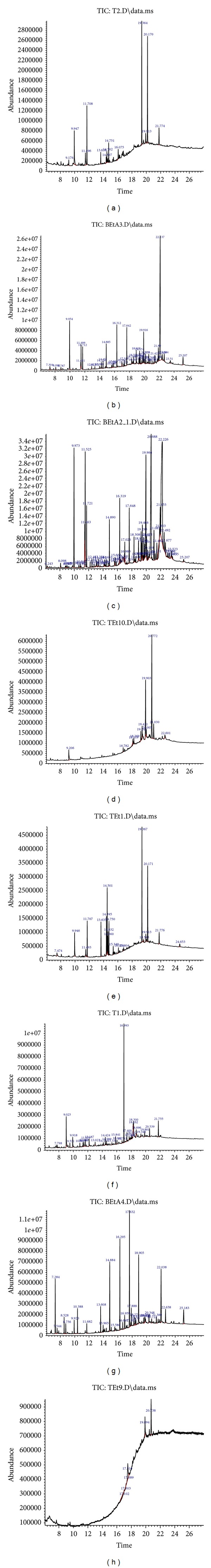
GC-MS total ion chromatogram (TIC) showing the retention time of the compounds identified from the fractions.

Though we proceeded to determine the possible compounds responsible for the observed activities in these fractions, Table 6 gives only the compounds identified in BEtA4, which had the best activity. However, volatile compounds identified in the fractions are those mostly found in food and medicine which are grouped into family of compounds such as hydrocarbon (cholest-2-eno[3,2-a]naphthalene, **4**′**-dimethylamino**; 0.2% TP; BEtA3), alcohol (9-O-pivaloyl-N-acetylcolchinol; 17% TP; TEt1), carbonyls (6-phenylamino-1H-pyrimidine-2,4-dione; 2.7% TP; BEtA3), acids (isopimaric acid TMS; 0.1% TP; BEtA2), esters (stigmasta-5,22-dien-3-ol, acetate, (**3.beta.,22Z**); 0.8% TP; T1), ethers (1-monolinoleoylglycerol trimethylsilyl ether, 2.1% TP, BEtA3; beta.-Sitosterol trimethylsilyl ether, 8.8% TP, BEtA2), acetals (cyclic 3,23-acetal with acetone, 0.2% TP, BEtA2; 3,4-dimethyl-1-dimethyl(allyl)silyloxycyclohexane, 0.9% TP, BEtA3), halogens (3-bromomethyl-3,6,6-trimethyl-cyclohexene; 2.6% TP; T2), amides (N-methyl-1-adamantaneacetamide; 3.4% TP; TEt10), phenols (phenol, 4-chloro-6-iodo-2-(4-iodophenyliminomethyl) and silane, trimethyl[5-methyl-2-(1-methylethyl)phenoxy]; 0.9% TP; BEtA3), bases (19-Norpregna-1,3,5(10)-trien-20-yn-2-amine, 3,17-bis[(trimethylsilyl)oxy], (17.alpha.); 1.8% TP; BEtA4), ketones (1-(2-naphthyloxy)-4-nitroanthraquinone; 0.2% TP; BEtA2), furans (fructofuranosyl 2,3,4,6-tetrakis-O-(trimethylsilyl), 3.4% TP, T2; Furan, 2-(diphenylamino)-4-(morpholinocarbonyl)-5- (p-nitrophenyl), 1% TP, BEtA3 and BEtA2 (1.3% TP)), coumarins (**2H-1,3-dithiolo[4,5-c]coumarine, 2-dicyanomethylene-8-nitro**; 0.4% TP; BEtA3), and sulfur compounds (2-(4-methoxy-phenyl)-7-oxo-7H-[1,2,4]triazolo[5,1-b][1,3]thiazine-5-carboxylic acid, methyl ester; 0.3 % TP; BEtA3) (Table 6 and Figure 1). 

## 4. Discussion

Resistance of microorganisms to currently available antimicrobial agents used in the treatment of infectious diseases requires new assessment. The antimicrobial susceptibility observed in the current study was concentration dependent. For example, 24.5 ± 3.5, 26.5 ± 2.1, and 32 ± 2.8 mm zones of inhibition were observed for *P. shigelloides* and 16.9 ± 0.2, 21.5 ± 2.1, and 26.4 ± 3.4 mm for *C. albicans* at 25, 50, and 100 mg/mL concentration of the extract, respectively. This is similar to the observation reported in our previous study of this extract against clinical strains of *H. pylori* [9]. Some of the bacteria and fungi species on the other hand were resistant to tetracycline. The incessant spread of multidrug-resistant pathogens has become a serious threat and a major public health concern worldwide, leading to the re-emergence of previously controlled diseases, which contributes extensively to the high incidence of opportunistic and chronic infection cases all over the world [3, 8, 10, 11]. 

The extract had MIC and MLC values of 0.15625 and 0.3125 mg/mL, respectively, against *P. shigelloides*, a Gram-negative bacterium that causes gastroenteritis. *P*.* shigelloides* has mostly been isolated from freshwater fish, shell fish, cattles, goats, swine, cats, dogs, monkeys, and snakes; and shares similar antigens with *S. sonnei* which has been implicated in dysentery [19]. *Salmonella Typhimurium* mostly found in the intestinal lumen has been etiologically linked to diarrhea and typhoid fever, while *H. pylori* is a major risk factor in peptic ulcer, gastritis, and gastric cancer in later life [9, 20, 21]. There was no discrepancy of activity observed with the extract in both Gram-negative and -positive (*S. pyogenes* and *S. aureus*) bacteria in the current study, an interesting finding which points to the fact that this plant could be used in the treatment of gastrointestinal related and other morbidities. 

The mechanism of antibiosis revealed that the extract was bacteriostatic/fungistatic against 3 organisms, bactericidal against 5, and was not considered ineffective against any of the organisms tested in our study (MLC/MIC_index_ ≥ 16). Fractions BEtA2, BEtA4, T1, and BEtA3 inhibited 90% of the test organisms (IC_90_) at 2.5 ± 0.8, 3.1 ± 2.3, 2.1 ± 0.8, and 3.8 ± 1.9 (mg/mL), respectively. Although it might seem reasonable that bactericidal drugs would have a preference to bacteriostatic drugs, the nature of infection is important in deciding which kind of drug to apply. For example, high concentrations of some bacteriostatic agents are also bactericidal, while low concentrations of some bactericidal agents are bacteriostatic [22]. Furthermore, in central nervous system infections, a bactericidal drug can discharge bacterial products that stimulate inflammation. Certain bacteriostatic drugs may be ideal in cases of streptococcal and clostridial gangrene, since they inhibit the production of the toxins that cause a great deal of the morbidity [13, 14, 22].

Although there is a dearth of information in the literature on the microbial and cytotoxic effect of the ethyl acetate extract of the stem bark of *P. africanum*, other solvents extract (methanol, acetone, dichloromethane hexane etc.) and plant parts have been reported to be active against some bacteria including *Campylobacter *spp., *Enterococcus fecalis*, *Staphylococcus aureus*, *Escherichia coli*, *Proteus mirabilis*, and *Helicobacter pylori* [9, 23, 24]. However, the effect of a drug on an organism is complex and involves interactions at multiple levels that cannot be predicted using biochemical assays. To understand this complexity, increase use of cell-based screening assays as more biologically relevant surrogates are being employed to predict the response of the organism. Moreover, in the drug discovery process, predicting cellular toxicity is essential and eukaryotic cell culture has been recognized as the model system of choice to get an approximation of toxicity [25]. 

The LD_50_ (lethal dose, 50%) indicates the quantity of extracts/compounds/drugs that, if administered to a population of organisms, will cause 50% of the organisms to perish. A high LD_50_ implies it would take a large quantity of the extract to cause a toxic response, while small LD_50_ values are highly toxic and could be dangerous. It was observed that the dose of the extract appeared to be more toxic after 24 hrs (log 1.92 ± 0.01; LD_50_ = 82.64 ± 1.40) of treatment than at 48 (log 2.15 ± 0.00; LD_50_ = 140.09 ± 1.33) and 72 hrs (2.08 ± 0.00; LD_50_ = 121.07 ± 0.92). Cell-based lethality assay is an indication of cytotoxicity, bactericidal and fungicidal activities, pesticidal effects, and various pharmacologic actions. The LD_50_ value obtained in the current study indicates that the extract has high pharmacological actions [26, 27]. The activity observed against bacteria, fungi, and human Chang liver cell may be ascribed to the compounds identified in the fractions of the extract. For example, phytosterols (e.g., silane,[[(3.beta.,24R)-ergost-5-en-3-yl]oxy]trimethyl) are recognized as save ingredients that lower blood cholesterol [28]. Gallic acid, **[(benzoic acid, 3,4,5-tris(trimethylsiloxy), trimethylsilyl)]** is a phenolic compound that helps to protect human cells against oxidative damage and cancer [29]. Lupeol, ferulic acid, and Hop-22(29)-en-3.beta.-ol acts as an antioxidant, anti-inflammatory, and antiulcerogenic agent [30]. 

## 5. Conclusions 

The ethyl acetate extract of *P. africanum* exhibited *in vitro* antibacterial (both Gram-negative and positive species) and antifungal activity. The major chemical compounds revealed by GC-MS analysis are believed to be responsible for the antimicrobial activity. However, since the extracts were toxic to the human Chang liver cells, we recommend that this plant extract should be used with caution, and further studies using *in vivo* (animal model) approach should be conducted to confirm this finding. 

## Figures and Tables

**Table 1:** Antimicrobial susceptibility test of the ethyl acetate extract compared with standard antibiotics.

Test bacteria and fungi	EAE*	EAE	EAE	Tetra.*	Amox.*
	25 mg/mL	50 mg/mL	100 mg/mL	30 *μ*g/mL	30 *μ*g/mL
*Helicobacter pylori *(ATCC 43526)	12.5 ± 0.7	14.25 ± 0.4	17.11 ± 0.2	0	0
*Pseudomonas aeruginosa* (ATCC 15442)	16 ± 1.4	16.5 ± 0.7	18 ± 1.4	7.5 ± 0.7	0
*Plesiomonas shigelloides* (ATCC 51903)	24.5 ± 3.5	26.5 ± 2.1	32 ± 2.8	26.7 ± 2.3	0
*Streptococcus pyogenes* (ATCC 49399)	17.6 ± 0.5	19.9 ± 1.2	24.1 ± 2.4	0	0
*Aeromonas hydrophila* (ATCC 35654)	17.5 ± 0.7	21.5 ± 2.1	24.9 ± 2.8	28.5 ± 2.1	0
*Staphylococcus aureus* (NCTC 6571)	12.8 ± 0.4	20.7 ± 0.4	25.6 ± 0.6	27.1 ± 1.3	0
*Shigella sonnei* (ATCC 29930)	18.6 ± 0.9	19.1 ± 0.2	21.9 ± 1.2	0	0
*Salmonella Typhimurium* (ATCC 13311)	19.1 ± 0.2	21.5 ± 2.1	24.1 ± 2.4	0	0
*Aspergillus flavus* (ATCC 204304)	0	0	0	0	0
*Aspergillus niger* (ATCC 16888)	0	0	0	0	0
*Candida albicans* (ATCC 2091)	16.9 ± 0.2	21.5 ± 2.1	26.4 ± 3.4	38.1 ± 0.2	36.9 ± 1.7
*Cryptococcus neoformans* (ATCC 66031)	7.5 ± 0.7	12.5 ± 0.7	14.25 ± 0.4	0	0

*EAE: ethyl acetate extract; Tetra.: tetracycline; Amox.: amoxicillin.

**Table 2:** Mechanism of antibiosis using MLC/MIC_index_ of the extract against test organisms.

Test bacteria and yeast	Ethyl acetate extract (mg/mL)			Tetracycline (*μ*g/mL)	
	MIC*	MLC*	MLC/MIC_index_	MIC	MBC*
*Helicobacter pylori *(ATCC 43526)	2.5	10	4	ND*	ND
*Pseudomonas aeruginosa* (ATCC 15442)	0.625	1.25	2	ND	ND
*Plesiomonas shigelloides* (ATCC 51903)	0.15625	0.3125	2	10	>10
*Streptococcus pyogenes* (ATCC 49399)	0.625	2.5	4	ND	ND
*Aeromonas hydrophila* (ATCC 35654)	2.5	5	2	10	>10
*Staphylococcus aureus* (NCTC 6571)	2.5	5	2	>10	>10
*Shigella sonnei* (ATCC 29930)	1.25	5	4	ND	ND
*Salmonella Typhimurium* (ATCC 13311)	1.25	2.5	2	ND	ND
*Candida albicans *(ATCC 2091)	5	>10	>2	10	>10
*Cryptococcus neoformans* (ATCC 66031)	10	ND	ND	ND	ND

*MIC: minimum inhibitory concentration; MLC: minimum lethal concentration; MBC: minimum bactericidal concentration; ND: not determined.

**Table 3:** Effective concentration and time interval on the percentage cell death.

Percentile	Probit			Log (dose)*		
	24 hrs	48 hrs	72 hrs	24 hrs	48 hrs	72 hrs
5	3.36	3.36	3.36	−0.75 ± 0.03	0.76 ± 0.01	1.05 ± 0.01
25	4.33	4.33	4.33	0.82 ± 0.02	1.58 ± 0.01	1.66 ± 0.01
50	5.00	5.00	5.00	1.92 ± 0.01	2.15 ± 0.00	2.08 ± 0.00
75	5.68	5.68	5.68	3.01 ± 0.01	2.72 ± 0.01	2.51 ± 0.00
95	6.65	6.65	6.65	4.59 ± 0.03	3.54 ± 0.01	3.12 ± 0.01

* Antilog which gives lethal dose in *μ*g/mL. Probit analysis NCSS 2007 used to determine log (dose), percentile, and probit values.

**Table 4:** Lethal dose at 50 percent reduction of the cell population.

Dose (*μ*g/mL)	Probit percent			Actual percent*			LD_50_ (*μ*g/mL)*		
	24 hrs	48 hrs	72 hrs	24 hrs	48 hrs	72 hrs	24 hrs	48 hrs	72 hrs
5	25.24	4.34	1.37	19.23	3.87	5.21	—	—	—
25	31.51	18.80	13.75	29.48	10.68	5.34	—	—	—
75	38.36	37.41	37.02	34.96	24.65	16.96	82.64 ± 1.40	—	—
125	45.59	47.66	50.88	55.96	65.03	70.68	—	140.09 ± 1.33	121.07 ± 0.92
250	51.03	61.70	69.21	60.12	71.17	79.41	—	—	—
500	62.54	74.33	83.68	61.85	72.16	82.88	—	—	—
1000	77.35	84.37	92.80	70.98	80.86	88.43	—	—	—

*LD_50_(50% of the cells have been killed); actual and probit percents were calculated using probit statistical analysis software “NCSS 2007”; *actual % = actual formulas (*n* is the number of cells in a group); —: not applicable.

**Table 5:** Inhibitory concentration (IC_50_ & _90_) of the eight isolated bioactive fractions (mg/mL).

Bacteria/yeast	T1	T2	TEt1	TEt9	TEt10	BEtA2	BEtA3	BEtA4
*A. hydrophila *								
IC_50_	0.391	3.125	3.125	1.563	1.563	1.563	1.563	1.563
IC_90_	1.563	ND*	ND	3.125	3.125	3.125	3.125	3.125
*H. pylori *								
IC_50_	6.25	3.125	6.25	3.125	6.25	1.563	ND	1.563
IC_90_	ND	ND	ND	ND	ND	3.125	ND	3.125
*P. aeruginosa *								
IC_50_	1.563	3.125	3.125	3.125	1.563	0.781	1.563	0.024
IC_90_	ND	ND	ND	ND	6.25	1.563	3.125	0.097
*P. shigelloides *								
IC_50_	1.563	3.125	3.125	3.125	3.125	1.563	1.563	0.781
IC_90_	3.125	ND	ND	ND	ND	3.125	6.25	1.563
*S. aureus *								
IC_50_	0.781	3.125	3.125	1.563	1.563	0.781	0.781	0.781
IC_90_	1.563	6.25	6.25	3.125	3.125	3.125	1.563	6.25
*S. pyogenes *								
IC_50_	0.781	3.125	1.563	3.125	3.125	0.781	1.563	0.391
IC_90_	3.125	ND	6.25	ND	ND	1.563	3.125	1.563
*S. sonnei *								
IC_50_	0.781	3.125	3.125	1.563	1.563	0.391	1.563	1.563
IC_90_	1.563	ND	ND	3.125	ND	1.563	3.125	6.25
*S. Typhimurium *								
IC_50_	0.391	3.125	3.125	1.563	3.125	0.781	3.125	1.563
IC_90_	1.563	ND	ND	3.125	ND	3.125	6.25	1.563
*C. albicans *								
IC_50_	1.563	3.125	ND	0.781	1.563	0.781	0.781	0.391
IC_90_	ND	6.25	ND	6.25	ND	1.563	1.563	1.563
*C. neoformans *								
IC_50_	1.563	3.125	6.25	1.563	ND	1.563	3.125	1.563
IC_90_	ND	ND	ND	3.125	ND	3.125	6.25	6.25

IC_50_	1.6 ± 1.7	3.125 ± 0	3.7 ± 1.6	2.1 ± 0.9	2.6 ± 1.6	1.0 ± 0.5	1.7 ± 0.9	1.0 ± 0.6
IC_90_	2.1 ± 0.8	6.25 ± 0	6.25 ± 0	3.7 ± 1.3	4.2 ± 1.8	2.5 ± 0.8	3.8 ± 1.9	3.1 ± 2.3

*ND: not determined.

**Table 6:** Identified compounds (twenty) from the BEtA4 fraction and their total percentage present.

BEtA4				
PK	RT	NIST05 library identified compounds	QM	TP (%)
1	7.2866	Benzoic acid, 3-methoxy-4-[(trimethylsilyl)oxy], trimethylsilyl ester	96	6.9
2	7.5432	Azelaic acid, bis(trimethylsilyl) ester	74	0.8
3	8.5248	Trimethylsilyl 3,5-dimethoxy-4-(trimethylsilyloxy)benzoate	99	2.0
4	8.7557	Cinnamic acid, 4-methoxy-3-(trimethylsiloxy), trimethylsilyl ester	99	1.5
5	9.9169	Hexadecanoic acid, trimethylsilyl ester	96	1.7
6	10.3852	Ferulic acid, trimethylsiloxy, trimethylsilyl ester	99	2.9
7	11.6811	Octadecanoic acid, trimethylsilyl ester	99	1.2
8	13.6057	Unknown	46	2.8
10	14.8823	Unknown	50	9.8
11	15.5816	Lanost-8-en-3-one	15	0.4
12	16.2937	Tetracosanoic acid, trimethylsilyl ester	98	10.7
13	16.6978	3-Oxo-9b-lanosta-7-en-26,23-olide	35	0.9
14	16.9609	Hexacosanoic acid, 2-[(trimethylsilyl)oxy], trimethylsilyl ester	22	2.6
15	17.6345	Lanosta-9(11),24-dien-3-ol, acetate, (3.beta.)	27	15.4
17	17.8911	19-Norpregna-1,3,5(10)-trien-20-yn-2-amine,3,17-bis[(trimethylsilyl)oxy], (17.alpha.)	43	1.8
18	18.2696	Unknown	38	1.9
21	18.9047	Unknown	3	9.8
28	22.0354	Hop-22(29)-en-3.beta.-ol	70	11.0
29	22.6577	Unknown	35	2.8
30	25.1853	Unknown	38	4.1

*PK: peak; RT: retention time; QM: quality march; TP: total percentage.
